# A rare cause of abdominal pain in pregnancy - mesenteric artery thrombosis and miscarriage in a 34-year-old patient

**DOI:** 10.1186/s12245-024-00661-x

**Published:** 2024-06-28

**Authors:** Dóra Melicher, Szabolcs Gaál, Tamás Berényi, Bánk Gábor Fenyves, Norbert Nagy, Péter Hegedűs, András Fülöp, Attila Szijártó, Csaba Varga

**Affiliations:** 1 Department of Emergency Medicine, Semmelweis University, Budapest, Hungary; 2 Department of Molecular Biology, Semmelweis University, Budapest, Hungary; 3 Medical Imaging Centre, Semmelweis University, Budapest, Hungary; 4 Department of Surgery, Transplantation and Gastroenterology, Semmelweis University, Budapest, Hungary

**Keywords:** Mesenteric ischemia, Emergency care, Early diagnosis, Factor VIII, Pregnancy

## Abstract

**Background:**

Mesenteric arterial thrombosis is an extremely rare thrombotic event, especially during pregnancy, that can cause rapid fatal consequences unless the patient receives early definitive treatment.

**Case presentation:**

We report the case of a 34-year-old female presenting in her seventh week of gestation with severe abdominal pain who was promptly diagnosed with mesenteric artery occlusion amidst incipient miscarriage. The patient underwent a successful mesentery artery embolectomy, recovered and was later diagnosed with elevated factor VIII activity.

**Conclusion:**

The diagnosis of mesenteric ischemia should be considered in pregnant women presenting with severe abdominal pain and any prior predisposing factors. Our case highlights the pivotal role of the emergency physician in maintaining a high index of suspicion coupled with timely and determined action. The prognosis of this high mortality condition depends on prompt diagnosis, early definite management and successful multidisciplinary cooperation.

## Background

Acute abdominal pain is one of the most common chief complaints for patients presenting to the Emergency Department (ED) [1]. Abdomino-pelvic pain during pregnancy is a frequent cause of ED presentation, acute pain can have a wide range of causes, including obstetric as well as non-obstetric origins, posing a formidable diagnostic and therapeutic challenge [2]. Non-obstetrical abdominal emergencies occur in one out of 500–700 pregnancies and may involve gastrointestinal, gynecological, urological, vascular and traumatic etiologies [3].

Acute mesenteric ischemia is a vascular emergency with a very high mortality rate [4–6]. Many of the signs and symptoms associated with acute mesenteric ischemia are common to other abdominal pathologies [6].

Acute intestinal ischemic disorder can be categorised into arterial occlusive mesenteric ischemia (AOMI), mesenteric venous thrombosis (MVT) and nonocclusive mesenteric ischemia [7]. According to epidemiology, acute arterial occlusion of the mesenteric artery is more common (incidence rate of 68%) than mesenteric venous thrombosis (incidence rate of 16%) [5]. Cases of arterial origin are generally associated with elderly people, cardiovascular diseases, atherosclerosis, atrial fibrillation, and cardiogenic emboli [8, 9]. Mesenteric venous thrombosis is associated with a hypercoagulable state; it is reported to have a greater occurrence in women of reproductive age with a history of repeated abortions, coagulopathies and autoimmune disorders [8, 10, 11].

Mesenteric ischemia associated with pregnancy is extremely rare. Cases of mesenteric vein thrombosis were attributed to the physiology of pregnancy itself, where there is an increase in factors VII and VIII, factor C and fibrinogen, which cause a hypercoagulable state [8, 12, 13]. Cases of mesenteric artery thrombosis during pregnancy have only been reported twice in literature, both in association with antiphospholipid syndrome [14, 15].

## Case presentation

A 34-year-old woman, 7 weeks pregnant, was brought to our emergency department (Department of Emergency Medicine, Semmelweis University) by ambulance, complaining of diffuse abdominal pain, back pain, chills and vaginal bleeding. On further questioning she stated that the severe pain started that day 40 min earlier; it was worse in the upper abdomen and around the belly, radiated to the back and was accompanied by chills. She stated the pain was unrelated to food intake. Her vaginal bleeding started in the ambulance car, where she also vomited once. She said that she was generally fine the days before, although she had some nausea and vomited twice with a minimal amount of blood noticed in it the day before. The patient had mild dyspnea. Her medical history was notable for deep vein thrombosis, which occurred twice, both after delivering her two children in 2006 and 2016, respectively. That time, her thrombophilia essay detected no abnormalities. Besides, her medical history included hypothyroidism, hysteroscopic metroplasty, one missed abortion and one pregnancy terminated by surgical method.

Physical examination revealed a soft abdomen, with diffuse abdominal and marked epigastric and periumbilical tenderness, accompanied by vaginal bleeding. Arterial blood gas analysis showed normoxia, normocapnia, normal pH and electrolyte levels; lactate level was mildly elevated (1.4 mmol/L).

While IV. analgesia and fluid therapy were initiated, an urgent gynecological consultation was organised 20 min after her arrival, revealing a live fetus by transvaginal ultrasound; no acute obstetric issues were identified.

Laboratory tests showed elevated inflammatory values (C-reactive protein: 21.6 mg/L, white blood cell count: 21 G/L), and D-dimer was 1.73 mg/L.

Considering the patient’s past medical history and present symptoms, including back pain, unsure dyspnea and specifically the two deep venous thromboses in association with her past pregnancies, echocardiography was organised, followed by a Doppler venous ultrasound of the low extremities. Echocardiography showed no right heart strain or other abnormalities, and no evidence of deep venous thrombosis was revealed through Doppler ultrasound. An abdominal ultrasound was also carried out to investigate any potential abdominal inflammatory process. It stated that the vermiform process could not be investigated, and there was no evidence of ileocecal inflammation or pathological abnormalities in other localisations.

**Fig. 1:**
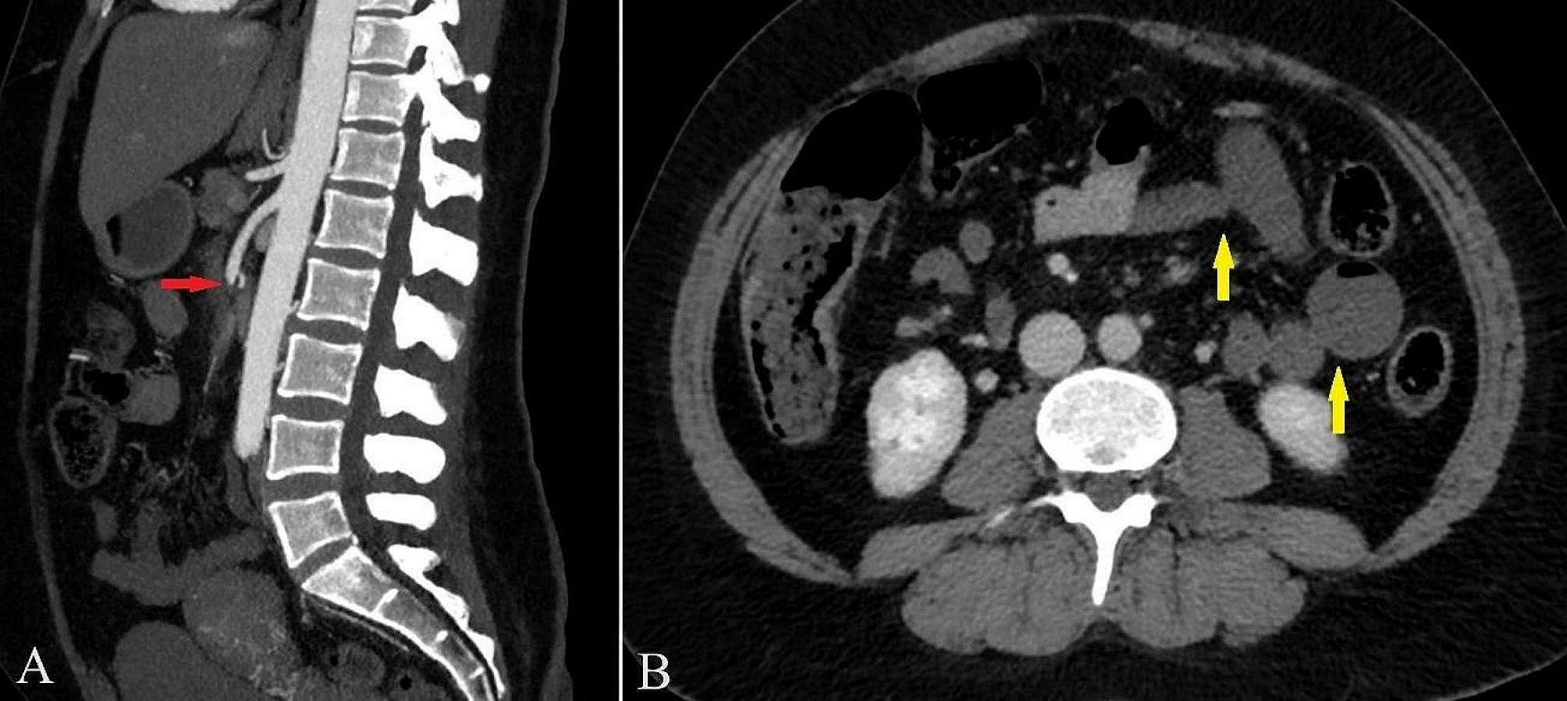
(**A**) Filling defect within the superior mesenteric artery approximately 4 cm distal to its origin consistent with occlusion (red arrow) on the sagittal maximum intensity projection (MIP) reconstruction CTA images. (**B**) The occlusion resulted in (**B**) perfusion changes: lack of enhancement and mild dilatation of the jejunum (yellow arrows) on the portal venous phase (Reference: Semmelweis University – Medical Imaging Centre)

Regardless of the repeated dose of IV non-opioid analgesics (1 gram of paracetamol repeated by 1 additional gram after an hour), the patient continued to complain of severe diffuse abdominal pain, and vaginal bleeding recurred again in the fourth hour of her ED observation. Therefore, the patient was transported for the second time to the Obstetric Department for gynecological consultation, where the repeated transvaginal ultrasound this time revealed an irregular ovarian sac and the fetus showing no signs of life. Incipient miscarriage was established. The patient was informed about the miscarriage, and she was administered IV oxytocin by the gynecologist to speed up the process. Right after that, an urgent abdominal computer tomography angiography (CTA) was organised, specifically interrogating any signs of mesenteric ischemia, while IV opioid analgetics (50 micrograms of fentanyl) were also administered. The scan confirmed that the superior mesenteric artery was occluded 4 cm after its origin (Fig. 1a). Consequently, intestinal pouches with residual contrast in the middle and distal thirds of the jejunum were described, while no sign of intestinal necrosis was seen (Fig. 1b).

**Fig. 2:**
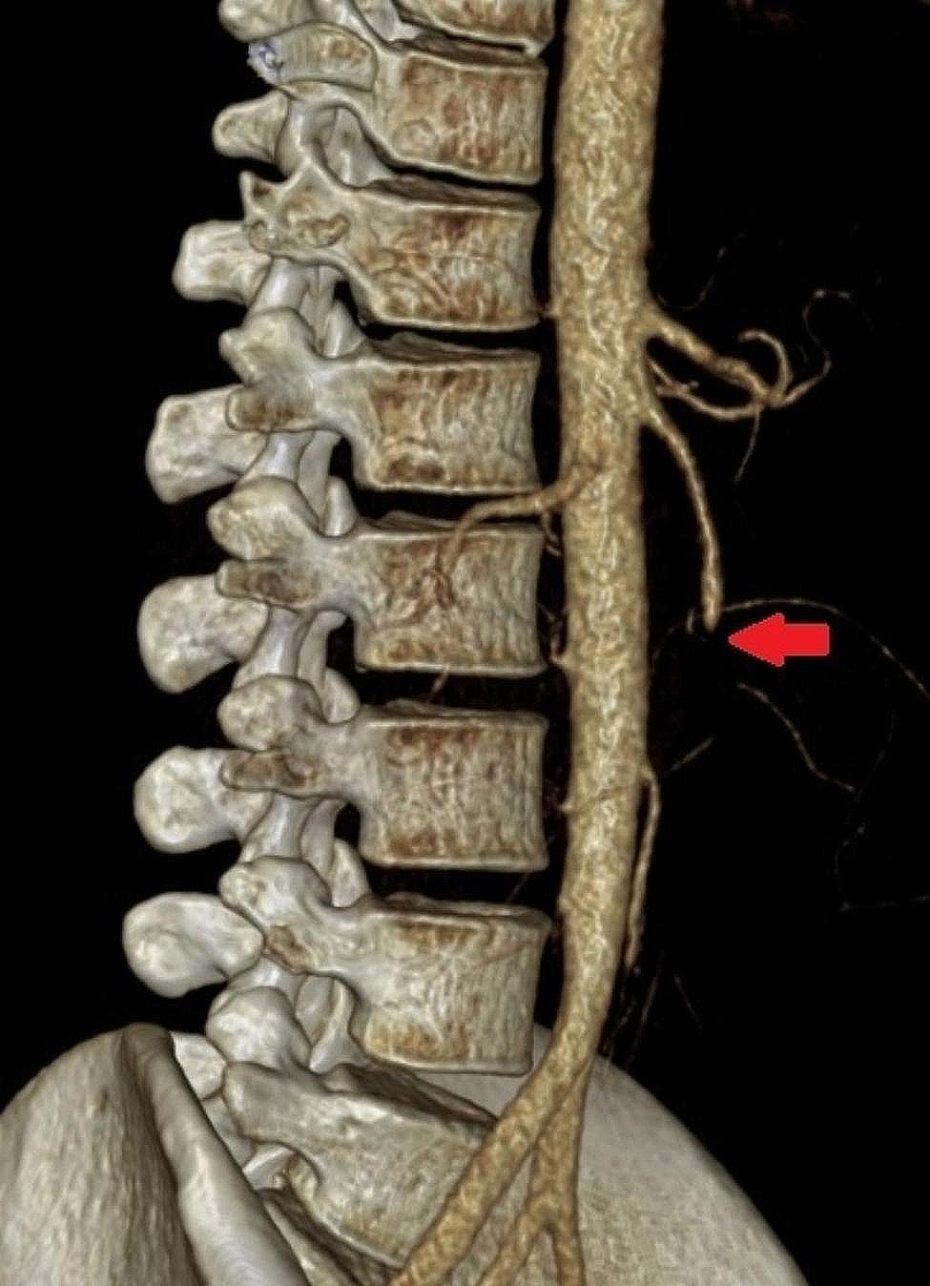
3D reconstruction of the CTA showing SMA occlusion (Reference: Semmelweis University – Medical Imaging Centre

Consultation with interventional radiologist, vascular surgeon, general surgeon and gynecologist were carried out right away simultaneously, resulting in the decision of acute surgical intervention. The patient was transferred to the Department of Surgery, where a superior mesenteric artery thrombectomy was performed the same evening (Fig. 2). The operation was done from median laparotomy with the joint contribution of vascular and general surgery, and curettage was also performed with gynecological cooperation. The second look laparotomy the next day revealed an intact small bowel and good pulsation in the superior mesenteric artery.

**Fig. 3:**
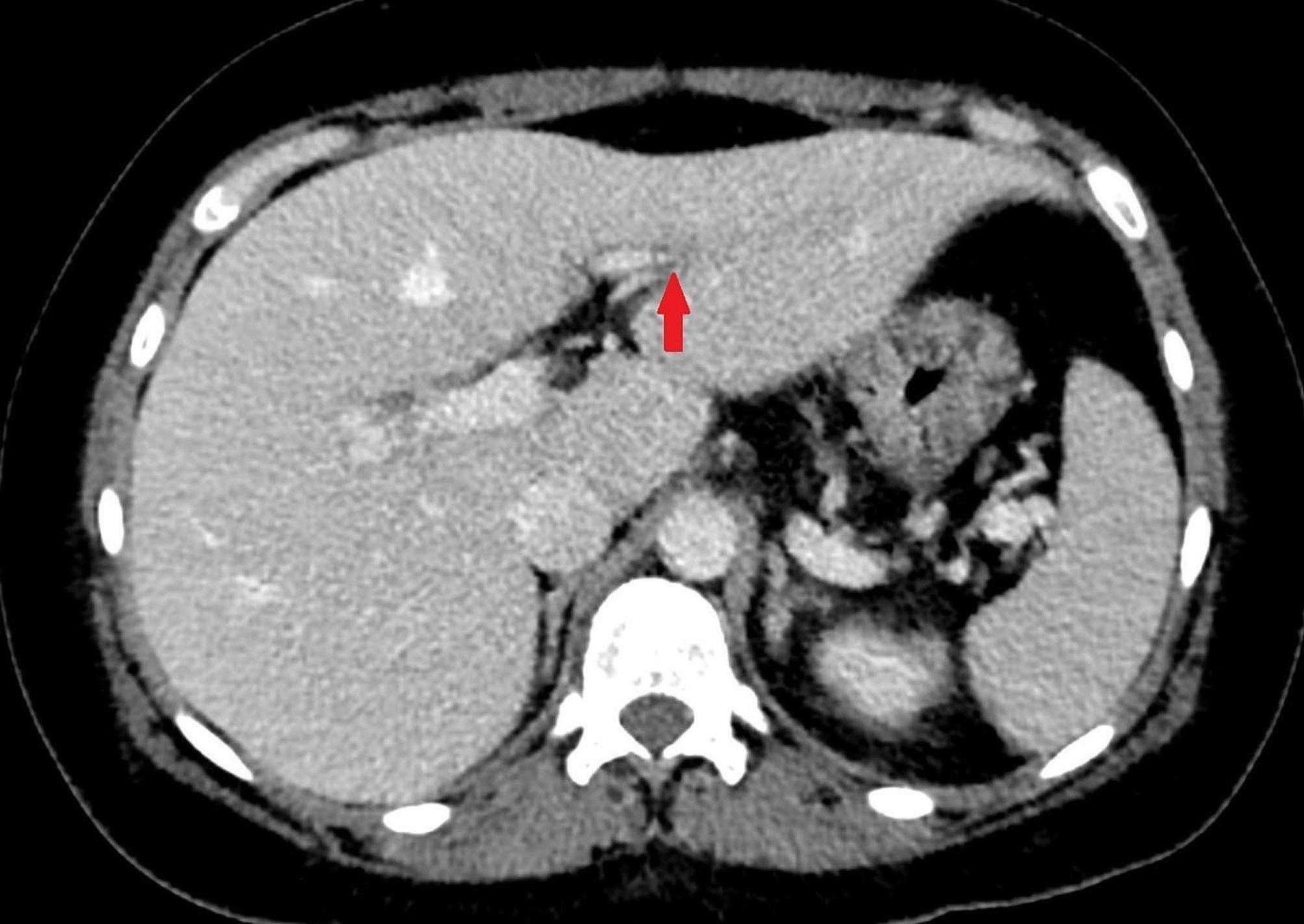
A faint hypodense filling defect is seen in the S.III. segmental portal venous branch in keeping with segmental portal vein thrombosis (Reference: Semmelweis University – Medical Imaging Centre

On the seventh day of her hospitalization at the Department of Surgery, follow-up CT scan was performed because the patient developed abdominal complaints. The scan described no evidence of surgical complication, but novum portal vein segmental thrombosis was seen, requiring no other therapy apart from therapeutic dose of LMWH (Fig. 3). Due to recurrent febrile status, a repeated gynecological consultation recommended combined antibiotic therapy in view of fever and elevated inflammatory parameters. Following therapy the patient became afebrile and no other signs of complications were observed. She was discharged home after 13 days of hospital stay and recovered.

**Fig. 4:**
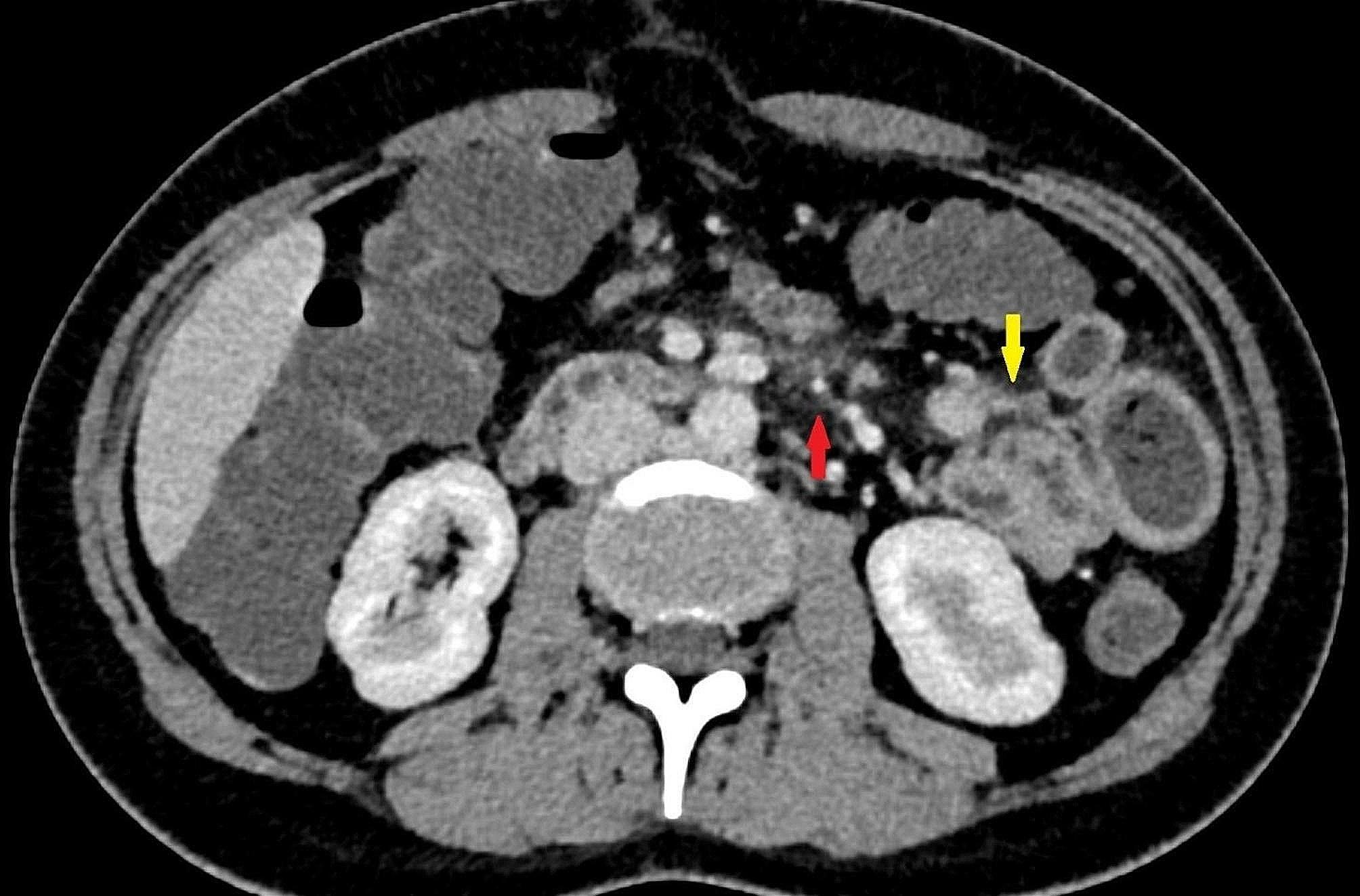
At 6-month follow-up examination on portal venous phase, hyperenhancing small bowel loops with thickened bowel wall (yellow arrow) are evident at the location of prior ischemic changes. Additionally, mild surrounding fat stranding and discernible lymphadenopathy (red arrow) are noted (Reference: Semmelweis University – Medical Imaging Centre)

After the surgical procedure, the patient underwent numerous follow-up examinations utilizing CT and MR imaging modalities without any sign of early or late complication. However subsequent imaging demonstrated persisting small bowel reperfusion injury changes at the site of prior ischaemic alterations (Fig. 4) [16, 17].

Thrombophilia test carried out three months later revealed elevated factor VIII levels. In addition, a JAK2 p.V617F mutation were identified with a variant allele frequency of 0.009%. According to the expert opinion, based on the low variant allele frequency, the clinical relevance of this mutation could be regarded questionable. Due to recurrent thrombotic events, the patient was recommended prolonged anticoagulation treatment.

## Discussion

Acute abdominal pain in pregnancy poses a unique diagnostic and therapeutic challenge. The pain can occur due to obstetric factors as well as for causes that are unrelated to pregnancy [2].

Aside from obstetric causes, abdominal pain can arise from gastrointestinal disease (appendicitis, cholecystitis, bowel obstruction, hepatic or splenic rupture), vascular pathology (splenic aneurysm, aortic aneurysmal dissection), gynecological pathology (aseptic necrosis of a uterine fibroid, ovarian or tubal torsion), urological pathology (nephrolithiasis with renal colic, pyelonephritis), or trauma [3].

Our patient presented with a leading complaint of severe, diffuse abdominal pain for 40 min and vaginal bleeding. We considered the above mentioned potential differential diagnoses and by timely sequential examinations we managed to rule them out. Obstetric consultation including transvaginal ultrasound excluded obstetric or gynecological causes, abdominal ultrasound supported with laboratory results proved no gastrointestinal or urological pathology, while doppler venous ultrasound and echocardiography showed no abnormalities either. As more frequent pathologies were ruled out and the intense pain of the patient persisted even following IV analgetic therapy, mesenteric ischemia was raised as a potential underlying pathology. Even in the setting of reoccurred vaginal bleeding and repeated transvaginal ultrasound establishing incipient miscarriage, the suspected differential diagnosis of mesenterial ischemia was maintained and prompted us to obtain abdominal CTA.

This case shows that acute mesenteric ischemia, including mesenteric artery occlusion should be considered in a pregnant woman with a leading complaint of abdominal pain and any prior thrombotic event. In the presented case previous deep vein thrombosis, gravidity as a hypercoagulable state, slightly elevated lactate level, persistent, severe abdominal pain, and sequential rule out of more common differential diagnoses urged us to investigate a possible mesenteric ischemia and therefore perform an abdominal CTA.

Emergency physicians have critical role in promptly identifying crucial warning signs and applying complex thinking and evaluation even in extremely challenging clinical settings. Only by maintaining a high clinical suspicion and appropriate modality of imaging can facilitate early diagnosis and consequently lifesaving final management. Systemic approach is required for an accurate and timely diagnosis of potentially life-threatening conditions. Pain out of proportion to physical examination should always be considered a warning sign of potentially serious pathology and prompt advanced imaging. The presented case highlights the crucial aspect of multidisciplinary cooperation in a coordinated manner and conceivably provides insights for management strategies of similar scenarios in the future.

Acute mesenteric ischemia is an uncommon diagnostic and therapeutic emergency with high rate of intestinal failure and mortality [4, 6]. Mesenteric vein thrombosis in pregnant patients is extremely rare, so far less than 20 reported cases can be found [18–20]. However, mesenteric artery thrombosis is even more exceptional during pregnancy, our literature search revealed only two reported cases, both related with antiphospholipid syndrome [14, 15].

Elevated Factor VIII levels were associated with venous thromboembolism [21]. Lin et al. in 2015 published the first report of acute arterial occlusive mesenteric ischemia associated with an elevated level of factor VIII [7]. Later reports confirmed that high factor VIII levels can be considered a risk factor for thrombosis, with a greater impact on venous than on arterial thrombosis [22].

Since the thrombophilia essay done three months after the diagnosed mesenteric artery thrombosis of our patient showed elevated factor VIII levels, we can presume that it could have contributed to AOMI in her condition as well.

To the best of our knowledge we are the first to report a case of mesenteric artery thrombosis during pregnancy associated with elevated factor VIII activity. Our presented case might urge further research to elucidate potential causes of mesenteric artery thrombosis in pregnancy. Investigating risk factors including elevated factor VIII activity offers additional avenues for future research.

## Concslusion

Our case underlines the imperative of early diagnosis and management of mesenteric artery thrombosis in pregnancy. It also highlights the pivotal role of the emergency physician, since only high-index-of-suspicion coupled with timely and determined action can facilitate appropriate treatment. The prognosis of this high mortality condition depends on prompt diagnosis, early definite management and well-coordinated multidisciplinary cooperation.

## Data Availability

No datasets were generated or analysed during the current study.

## Abbreviations

AOMI: Arterial occlusive mesenteric ischemia
CTA: Computed Tomography Angiography
MVT: Mesenteric venous thrombosis
